# Decoding the Diverse Colours of Guillain-Barré Syndrome (GBS): A Case Report and Narrative Review of Atypical Miller Fisher-GBS Overlap Syndrome

**DOI:** 10.7759/cureus.111465

**Published:** 2026-06-25

**Authors:** Aliyah Aykac, Richard Oguntoye, Ahmet Ubur, Paul Bolaji

**Affiliations:** 1 Stroke Medicine, Dorset County Hospital, Dorchester, GBR; 2 Neurology, University Hospitals Birmingham NHS, Birmingham, GBR

**Keywords:** guillain-barré-like syndrome, guillain-barré syndrome, guillain-barré syndrome (gbs), miller fisher, miller fisher syndrome, miller fisher syndrome (mfs), miller fisher variant

## Abstract

The classic presentation of Guillain-Barré syndrome (GBS), as described in older medical textbooks, is that of a patient with a preceding history of diarrhoea who develops rapidly progressive, symmetrical quadriparesis with areflexia. These clinical features have often been deeply entrenched in the minds of physicians, such that deviation from this pattern frequently casts doubt on the diagnosis of GBS.

In contrast, it is widely recognised that GBS presents across a diverse spectrum of variants sharing a similar pathophysiology. The heterogeneity of GBS is driven by the different neural targets in different parts of the neural axis. This explains the diverse manifestations of GBS and alerts clinicians to focus on the hard and invariable signs of GBS rather than the other soft and variable features of GBS chameleon to ensure early diagnosis and prompt initiation of immunomodulatory treatment.

GBS presentation can range from the classic acute inflammatory demyelinating polyradiculoneuropathy (AIDP) to rarer variants such as Miller Fisher syndrome (MFS), acute pan-dysautonomia, Bickerstaff encephalitis, and even triple overlap syndromes involving Miller Fisher syndrome, GBS, and Bickerstaff encephalitis, among others, documented in the literature.

This case report and narrative review follows a 69-year-old Caucasian man who presented atypically and was diagnosed with Miller Fisher-GBS overlap syndrome. This article highlights an atypical presentation of a Miller Fisher-GBS overlap syndrome with right-sided weakness and severe back pain, ophthalmoparesis, and proximal limb weakness, before progressing to classical GBS with quadriparesis. Despite initial misdiagnoses of vasculitis and myasthenic crisis, a definitive diagnosis of GBS was eventually made, and the patient received prompt immunomodulatory treatment with a favourable outcome.

It underscores the importance of thorough neurological localisation, emphasising the need to build diagnostic frameworks on hard neurological signs rather than variable and inconsistent soft signs. Most importantly, it highlights the varied presentations of GBS and reviews the underlying immunopathogenesis and management of GBS variants.

## Introduction

Guillain-Barré syndrome (GBS) is a rare, immune-mediated disorder affecting the peripheral nervous system. It encompasses a spectrum of related variants with differing phenotypes, electrophysiological findings, and prognostic outcomes [1]. The worldwide annual incidence of GBS is approximately 1-2 per 100,000. Of these, approximately 1%-7% represent Miller Fisher syndrome (MFS), with Miller Fisher-GBS overlap syndrome being rarer still. It is widely recognised, however, that the incidence of MFS is higher in certain populations, particularly in Asian countries, where its prevalence may be as high as 25% of GBS cases [2].

The true frequency of the classic acute inflammatory demyelinating polyradiculoneuropathy (AIDP) subtype varies widely, accounting for approximately 30%-80% of GBS cases globally. Other recognised presentations include acute motor axonal neuropathy (AMAN) and acute motor-sensory axonal neuropathy (AMSAN), as well as rarer variants such as Miller Fisher syndrome, acute ataxic neuropathy, pharyngeal-cervical-brachial variant, multiple cranial neuropathy variant, facial diplegia with paraesthesias, paraparetic variant, acute pan-dysautonomia, and Bickerstaff encephalitis.

There are no separate population-level incidence data for atypical Miller Fisher-GBS overlap syndrome, as it is rare, often misclassified, and frequently grouped under MFS spectrum disorders and anti-GQ1b antibody-associated conditions.

GBS and its variants are commonly triggered by viral or bacterial infections, particularly *Campylobacter jejuni*, which causes a diarrhoeal illness. Other recognised triggers include trauma, surgical procedures, certain medications, notably immune checkpoint inhibitors, and systemic illnesses.

Molecular mimicry underlies the immune-mediated attack on nerve roots and heavily myelinated nerve fibres, leading to the characteristic albuminocytological dissociation seen in cerebrospinal fluid (CSF) and, in some cases, positive antiganglioside antibodies. Nerve conduction studies performed after the first week typically demonstrate conduction block and loss of H-reflexes [3].

## Case presentation

A 69-year-old Caucasian man was admitted overnight to the stroke unit of a district general hospital in South West England with a constellation of symptoms, including acute-onset proximal right-sided weakness, moderate truncal ataxia, generalised headache, severe lower back pain, chest pain, weight loss, and horizontal diplopia. At presentation to the emergency department, he remained mobile but unsteady. A computed tomography (CT) scan of the head excluded intracranial haemorrhage. Owing to the severity of his pain, he was initially unable to provide a clear history.

The following morning, the stroke team reviewed the patient and noted right-sided ptosis with normal pupils, binocular diplopia due to right eye adduction palsy, bilateral proximal upper limb weakness (worse in the right arm), and right leg weakness. Distal power was preserved. Reflexes were reduced but present. Laboratory investigations are summarised in Table 1, which revealed an elevated erythrocyte sedimentation rate (ESR), with all other parameters within normal limits.

**Table 1:** Admission haematology and biochemistry Routine haematological and biochemical investigations performed on admission. An elevated ESR was the sole notable finding. Urine porphyrin/creatinine ratio was within normal limits, making porphyria-related motor neuropathy less likely. ESR: erythrocyte sedimentation rate, Hb: haemoglobin, CRP: C-reactive protein, TSH: thyroid-stimulating hormone, eGFR: estimated glomerular filtration rate

Investigation	Result	Reference range	Interpretation
Hb	163 g/L	130-180 g/L (male)/115-165 g/L (female)	Within range
Platelets	215 × 10⁹/L	150-400 × 10⁹/L	Within range
White cell count	6.6 × 10⁹/L	4.0-11.0 × 10⁹/L	Within range
Neutrophils	4.7 × 10⁹/L	1.8-7.5 × 10⁹/L	Within range
CRP	<1 mg/L	<5 mg/L	Within range
ESR	62 mm/hr	1-15 mm/hr (male)/1-20 mm/hr (female)	Raised
Magnesium	0.75 mmol/L	0.70-1.00 mmol/L	Within range
Phosphate	0.81 mmol/L	0.80-1.50 mmol/L	Within range
Vitamin B12	418 ng/L	200-900 ng/L	Within range
Folate	4.1 µg/L	3.0-20.0 µg/L	Within range
TSH	1.1 mU/L	0.35-5.50 mU/L	Within range
Free T4	20 pmol/L	9-25 pmol/L	Within range
Potassium	4.2 mmol/L	3.5-5.3 mmol/L	Within range
Urea	5.3 mmol/L	2.5-7.8 mmol/L	Within range
Sodium	135 mmol/L	133-146 mmol/L	Within range
eGFR	74 mL/min/1.73 m²	≥60 mL/min/1.73 m²	Within range
Creatinine	91 µmol/L	59-104 µmol/L (male)/45-84 µmol/L (female)	Within range
Glucose	5.4 mmol/L	4.0-7.8 mmol/L	Within range
Urine creatinine	12.2 mmol/L	4.0-18.0 mmol/L	Within range
Urine porphyrin/creatinine ratio	9 nmol/mmol	<22 nmol/mmol	Within range
Blood cultures	No growth after 48 hours	No growth	Within range

The differential diagnosis at this stage included myasthenia gravis and vasculitis, notably giant cell arteritis, with GBS considered only a remote possibility. Further investigations were undertaken, with results detailed in Tables 2-4. Multiple imaging modalities were employed, and Table 5 summarises all imaging reports. Figure 1 shows the CT of the head, which demonstrated no acute intracranial abnormality, and Figure 2 presents the MRI of the brain, which was also unremarkable. Figure 3 displays the MRI of the cervical spine, revealing no structural or inflammatory pathology. Figure 4 illustrates the magnetic resonance angiography (MRA) findings, which showed no evidence of large‑vessel vasculitis. Figure 5 depicts the shoulder X‑ray performed due to the patient’s shoulder weakness, and this too was normal. Vasculitic screening returned negative, and rheumatology input corroborated that the clinical presentation was not consistent with vasculitis.

**Table 2:** Infectious and vasculitic serology Serology performed to exclude infectious and vasculitic aetiologies. All results were negative or within normal limits. HIV: human immunodeficiency virus, MPO: myeloperoxidase, ANCA: anti-neutrophil cytoplasmic antibody, PR3: proteinase 3

Investigation	Result	Reference range
Syphilis antibody	Not detected	Not detected
Lyme disease IgG antibody	Not detected	Not detected
Lyme disease IgM antibody	Not detected	Not detected
HIV-1/2 antigen/antibody	Not detected	Not detected
MPO-ANCA	Negative (0.5 IU/mL)	0-3.49 IU/mL
PR3-ANCA	Negative (<0.6 IU/mL)	0-1.99 IU/mL

**Table 3:** Autoimmune and paraneoplastic antibody panel Serum autoimmune and paraneoplastic antibody screen performed to exclude autoimmune encephalitis and neuromuscular junction pathology. All results were within normal limits or negative. VGKC: voltage-gated potassium channel

Investigation	Result	Reference range
Anti-AMPA 1	Negative	Negative
Anti-AMPA 2	Negative	Negative
Anti-GABA-B	Negative	Negative
Western blot for paraneoplastic antibodies	Negative	Negative
Anti-VGKC antibodies	Negative	Negative
Acetylcholine receptor antibody	0.37 nmol/L	0-0.89 nmol/L

**Table 4:** CSF analysis Lumbar puncture performed as part of the diagnostic workup. Notably elevated CSF protein with normal cell count is consistent with cytoalbuminous dissociation, a recognised feature of Guillain-Barré syndrome. CSF: cerebrospinal fluid

Investigation	Result	Reference range	Interpretation
CSF glucose	3.4 mmol/L	2.22-3.89 mmol/L	Within range
CSF protein	1.02 g/L	0.15-0.45 g/L	Raised
CSF lactate	1.7 mmol/L	1.1-2.4 mmol/L	Within range
CSF microscopy	Unremarkable	0 white cells, no organisms	Within range

**Table 5:** Neuroimaging summary Neuroimaging performed to exclude structural, vascular, and compressive aetiologies. All imaging modalities were unremarkable. CT: computed tomography, MRI: magnetic resonance imaging, MRA: magnetic resonance angiography

Investigation	Report
CT of the head	No features to suggest acute infarction or haemorrhage.
MRI of the brain	There is microhaemorrhage in the right inferior parietal left/posterior temporal lobe. No similar haemorrhages elsewhere. No evidence of acute infarct. No large intra- or extra-axial bleed.
MRI of the cervical spine	Normal signal is noted in the spinal cord with no evidence of myelopathy. No focal lesion or pathological fracture was demonstrated. Multiple bilateral perineural sheath cysts at lower cervical levels. At C4-5, mild right foramen stenosis due to disc osteophyte complex; however, no significant canal stenosis at this level. Remaining levels are unremarkable. Disc degenerative changes as described above.
MRA of the head	No major stenosis or occlusion in the intracranial circulation. No features to suggest vasculitis. No aneurysm or vascular malformation. No significant intracranial abnormality.
X-ray of the shoulder	No fracture or dislocation seen. The acromioclavicular and glenohumeral joints appear to be normally located. The subacromial space is within normal limits. No calcified loose bodies seen within the joint space, and no evidence of calcific tendinopathy. Imaged lungs and pleural spaces appear clear.

**Figure 1:**
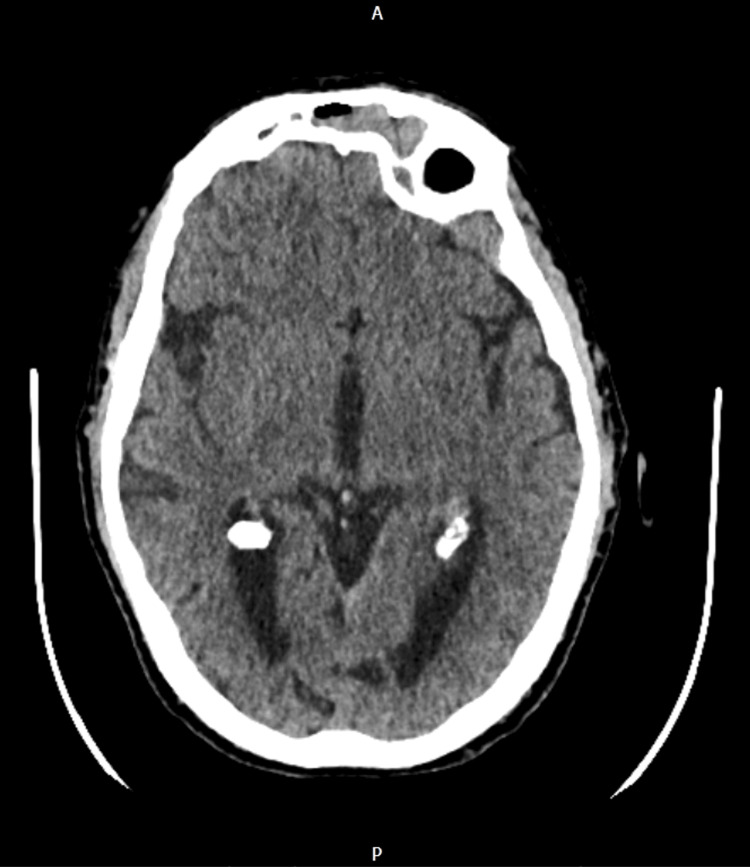
Axial non‑contrast CT of the head demonstrating normal brain attenuation, preserved grey-white differentiation, and normal ventricular and extra‑axial spaces with no acute intracranial abnormality CT: computed tomography

**Figure 2:**
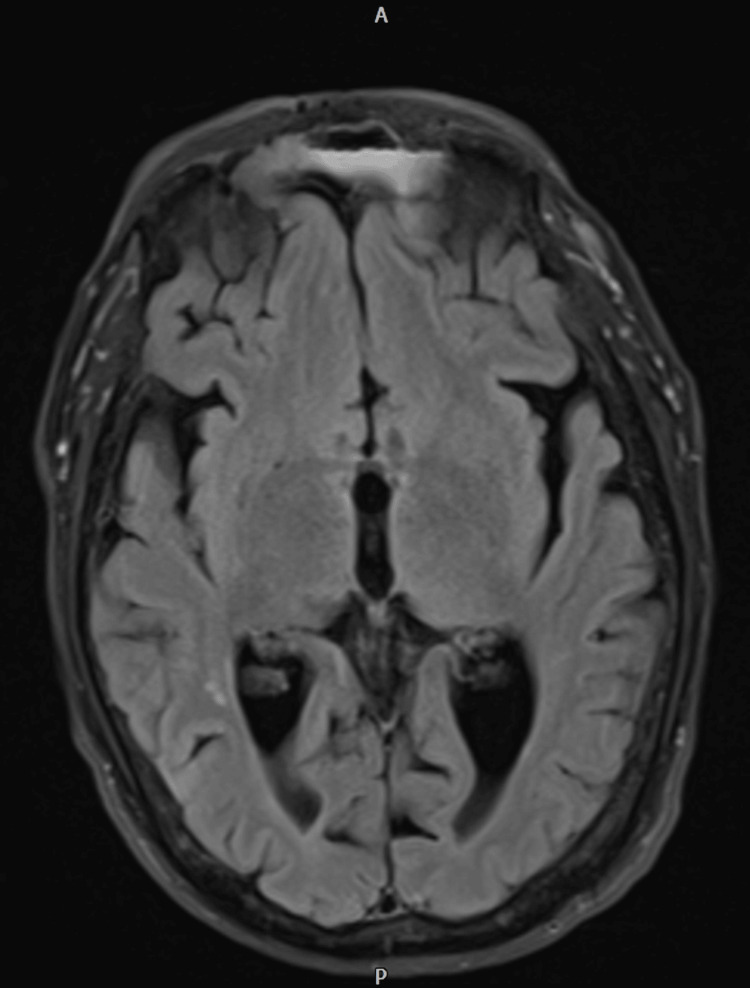
Axial MRI of the brain T2-weighted demonstrating normal parenchymal signal, preserved grey-white differentiation, normal ventricular and extra‑axial spaces, and no diffusion restriction or acute intracranial abnormality MRI: magnetic resonance imaging

**Figure 3:**
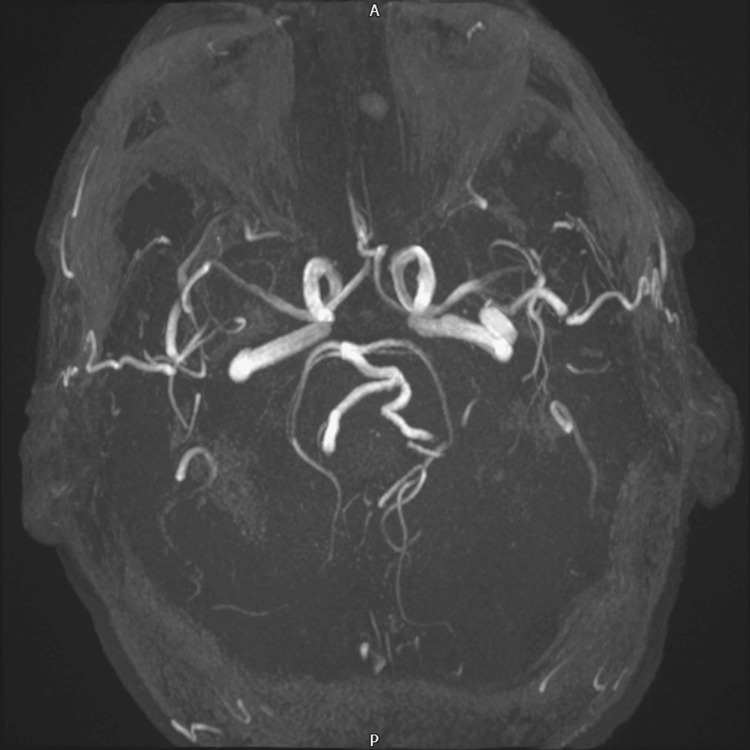
Normal intracranial MRA demonstrating patent anterior and posterior circulations with no stenosis, aneurysm, or vascular malformation MRA: magnetic resonance angiography

**Figure 4:**
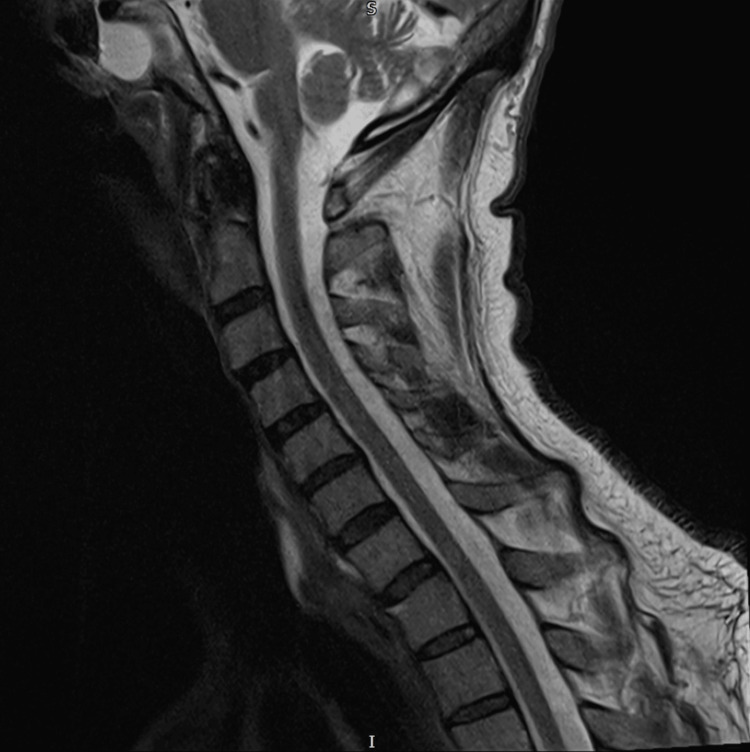
MRI of the cervical spine The spinal canal is capacious throughout. Normal signal is noted in the spinal cord with no evidence of myelopathy. No focal lesion or pathological fracture was demonstrated. MRI: magnetic resonance imaging

**Figure 5:**
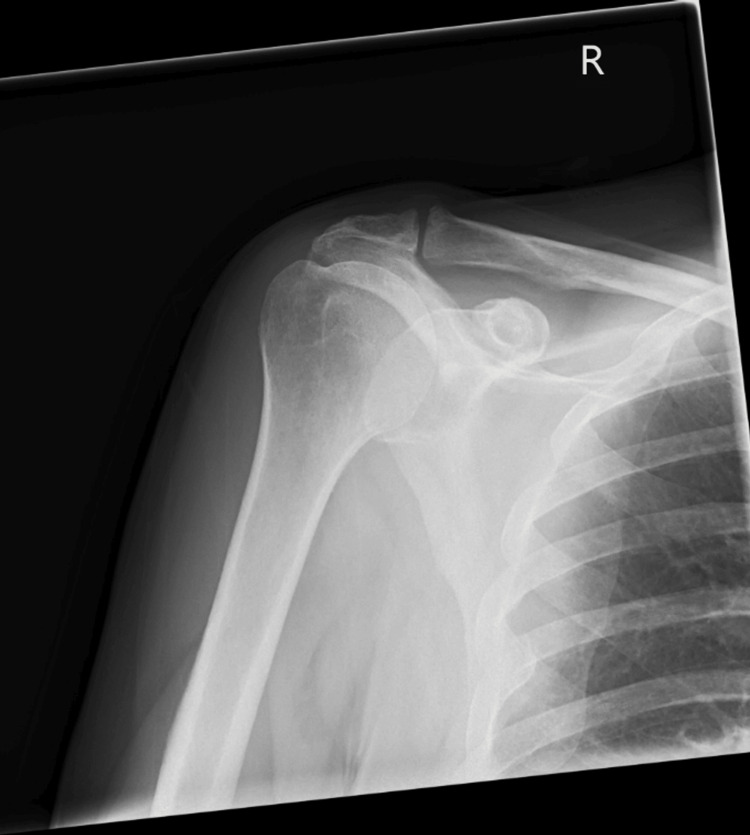
X-ray of the right shoulder The acromioclavicular and glenohumeral joints appear to be normally located. The subacromial space is within normal limits. No calcified loose bodies were seen within the joint space, and there was no evidence of calcific tendinopathy.

The patient was empirically treated for a presumed myasthenic crisis with pyridostigmine and prednisolone. Intravenous immunoglobulin (IVIG) was prescribed for five days, but initially administered for only one day. Spirometry was monitored four times daily.

Three days later, the patient was reassessed by a second stroke consultant, who noted progression of symptoms despite treatment with steroids and pyridostigmine. By this point, the patient had developed bilateral adduction palsy with right-sided ptosis and mild bilateral facial weakness. There was no dysphagia, although cough strength was reduced. Fatigability testing was negative.

Neurological examination at this stage revealed quadriparesis, with proximal power graded 2/5 in all four limbs and distal power approximately 4/5. Reflexes, including biceps, triceps, supinator, knee, and ankle jerks, were globally absent even with reinforcement. Plantar responses were flexor. A suspended, patchy sensory loss was noted across both upper and lower limbs without a clear sensory level. On the basis of these findings, a provisional diagnosis of Miller Fisher-GBS overlap syndrome was made.

Prednisolone and pyridostigmine were discontinued immediately, and a full five-day course of IVIG was administered. Acetylcholine receptor antibodies were negative. Serological testing for human immunodeficiency virus (HIV), cytomegalovirus (CMV), Epstein-Barr virus, Lyme disease, and syphilis were all negative. Lumbar puncture demonstrated elevated cerebrospinal fluid protein with a normal cell count, consistent with albuminocytological dissociation. CSF viral PCR and cultures were negative.

Nerve conduction studies, as detailed in Tables 6-8, were performed approximately 10 days after symptom onset and revealed motor nerve abnormalities affecting the right upper limb and both lower limbs. Mild early demyelinating features were identified in the lower limbs, with mild axonal abnormalities in the right upper limb, predominantly in a proximal distribution. No pathological temporal dispersion was noted. Taken together, these findings supported a mixed demyelinating and axonal motor polyradiculoneuropathy consistent with a GBS variant. Porphyria-related motor neuropathy was considered but excluded by a normal urinary porphobilinogen level.

**Table 6:** Motor nerve conduction studies CMAP: compound muscle action potential, EDB: extensor digitorum brevis, AH: abductor hallucis, APB: abductor pollicis brevis, ADM: abductor digiti minimi

Nerve	Side	Distal latency (ms)	Normal distal latency (ms)	CMAP amplitude (mV)	Normal CMAP (mV)	Conduction velocity (m/s)	Normal CV (m/s)
Common peroneal (EDB)	Right	Prolonged	<6.5	Normal	>2	Normal	>44
Common peroneal (EDB)	Left	Prolonged	<6.5	Normal	>2	Normal	>44
Tibial (AH)	Right	Severely prolonged	<6.1	Normal	>4	-	>41
Tibial (AH)	Left	Severely prolonged (> right)	<6.1	Normal	>4	-	>41
Median (APB)	Right	Severely prolonged	<4.4	Severely attenuated	>4	Normal	>49
Ulnar (ADM)	Right	Normal	<3.6	Attenuated	>6	Normal	>49

**Table 7:** Sensory nerve conduction studies SNAP: sensory nerve action potential, CV: conduction velocity

Nerve	Side	SNAP amplitude	Normal SNAP amplitude (µV)	CV (m/s)	Normal CV (m/s)
Tested sensory nerves (upper and lower limbs)	Right	Within normal limits	>10	Within normal limits	>45

**Table 8:** Needle EMG findings Peripheral neurophysiology demonstrated motor nerve abnormalities affecting the right upper limb and bilateral lower limbs. Early demyelinating features were identified in the lower limbs, with mild axonal abnormalities in the right upper limb (proximal > distal distribution). The overall pattern is consistent with a mixed early demyelinating and axonal motor polyradiculoneuropathy, representing a variant form of Guillain-Barré syndrome. EMG: electromyography

Muscle	Side	Spontaneous activity	Volitional activity	Interpretation
Deltoid	Right	Mild subacute denervating features	-	Mild motor denervation
Proximal upper limb muscles (other)	Right	None	Moderately-severely reduced	Reduced voluntary activation, no denervation
Lower limb muscles (proximal and distal)	Right	None	Normal	No significant abnormality

A visiting neurologist subsequently reviewed the patient and all investigation results, as summarised in Tables 1-8, and concurred with the diagnosis of GBS with an atypical Miller Fisher variant. The diagnosis fulfilled established diagnostic criteria for Guillain-Barré syndrome according to the European Academy of Neurology and Peripheral Nerve Society guidelines. The diagnosis in this case was based on the characteristic clinical presentation, cerebrospinal fluid findings, and electrophysiological evidence of acute inflammatory polyradiculoneuropathy.

Despite initial worsening to profound quadriparesis, the patient maintained airway function throughout. His predominant symptom remained severe back pain, which significantly disrupted sleep. Gabapentin was initially prescribed and subsequently switched to pregabalin, with additional PRN oxycodone. Pain control remained challenging and ultimately improved with the addition of duloxetine 60 mg daily.

The patient underwent multidisciplinary rehabilitation involving physiotherapy and occupational therapy. Over several weeks, his ptosis and ophthalmoparesis resolved, and limb strength improved to 4/5 proximally and 4+ distally. By seven weeks post-admission, he had progressed from hoist-assisted transfers to mobilising with a rollator frame and was transferred to a level 2B community rehabilitation hospital. The patient spent under a month in the rehabilitation hospital and was discharged home, undergoing further fortnightly rehabilitation as an outpatient. Six months post-presentation, the patient had made significant improvement and was mobilising independently and returning to playing the guitar.

## Discussion

This case illustrates an atypical presentation of GBS characterised by right-sided weakness and severe back pain. Associated diplopia, unsteadiness, and ptosis further complicated the diagnostic framework, with additional confounders including nausea and weight loss. Initial suspicion of vasculitis, prompted by an elevated ESR, was discounted following normal neuroimaging and rheumatology review. Serum angiotensin-converting enzyme (ACE) was normal, which does not support a diagnosis of sarcoidosis. Myasthenic crisis was considered, given the ophthalmoparesis and proximal weakness, but was later excluded by the presence of areflexia, lack of fatigability, and absence of response to pyridostigmine and steroids.

The rapid progression to quadriparesis with absent reflexes ultimately unveiled the underlying diagnosis of GBS. Importantly, GBS does not always present with the classical symmetrical ascending AIDP phenotype; rather, clinical manifestations vary according to the subtype. Knowledge of GBS variants, careful attention to hard neurological signs, and awareness of symptom evolution are therefore critical to reaching a timely diagnosis.

This case represents an atypical presentation of Miller Fisher-GBS overlap syndrome, which is itself a rare occurrence. It underscores the protean nature of GBS beyond the classic ascending phenotype, once considered pathognomonic of the condition.

Classification of GBS

GBS classification is often based on clinical and neurophysiological context. Classifying based on a neurophysiological template into demyelination or axonal picture might help in prognosticating the lesion [2]. A non-exhaustive list of GBS presentations is presented in Table 9.

**Table 9:** Classification of GBS variants This table was created by the authors. It was not reproduced or adapted from any external source; no permissions are required. GBS: Guillain-Barré syndrome, AIDP: acute inflammatory demyelinating polyradiculoneuropathy, AMAN: acute motor axonal neuropathy, AMSAN: acute motor-sensory axonal neuropathy, MFS: Miller Fisher syndrome, ICU: intensive care unit, AO: acute ophthalmoparesis, PCB: pharyngeal cervical brachial variant, BBE: Bickerstaff brainstem encephalitis, CNS: central nervous system

GBS variant	Core clinical features	Reflexes	Weakness pattern	Sensory features	Cranial nerve involvement	Associated antibodies	Notes/distinguishing points
Classic GBS (AIDP)	Ascending paralysis, areflexia, autonomic dysfunction	Absent	Symmetrical, ascending	Common (paraesthesia)	Facial nerve common	Nonspecific	Most common in Europe/NA; demyelinating
AMAN	Acute flaccid paralysis, rapid progression	Reduced/absent	Pure motor, often severe	Absent	Rare	Anti-GM1, GD1a	Axonal; common in Asia
AMSAN	Severe motor + sensory axonal neuropathy	Absent	Severe, diffuse	Prominent	Rare	Anti-GM1, GD1a	Poorer prognosis
Miller Fisher syndrome (classic)	Ophthalmoplegia, ataxia, areflexia	Absent	Minimal or none	Minimal	III, IV, VI	Anti-GQ1b (90%)	Favourable prognosis
Atypical Miller Fisher syndrome	Incomplete triad; asymmetric or unilateral ophthalmoplegia; limb weakness or sensory symptoms	May be reduced, absent, or preserved	Mild limb weakness may occur	May be present	Variable	Anti-GQ1b, GT1a, GD1b	Often under-recognised
MFS-GBS overlap	MFS features + limb weakness typical of GBS	Reduced/absent	Ascending weakness	Common	Frequent	Anti-GQ1b, Anti-GM1	Higher ICU/ventilation risk
AO	Isolated ophthalmoplegia without ataxia	Normal	None	None	III, IV, VI	Anti-GQ1b	Part of anti-GQ1b spectrum
PCB	Dysphagia, neck and arm weakness	Reduced	Upper limb predominant	Minimal	IX, X, VII	Anti-GT1a	Can mimic a stroke
BBE	Ophthalmoplegia, ataxia, impaired consciousness	Often preserved	Variable	Variable	Brainstem	Anti-GQ1b	CNS involvement
Pure sensory GBS	Sensory ataxia, pain	Absent	None	Prominent	Rare	Anti-GD1b	Rare; diagnostic challenge
Autonomic GBS	Dysautonomia predominant	Variable	Minimal	Minimal	Rare	Nonspecific	Can be life-threatening

Miller Fisher syndrome and its variants

Overlap syndromes involving MFS and GBS, as well as triple overlap syndromes with Bickerstaff encephalitis, have been described in the literature.

MFS itself is rare, accounting for 5%-10% of GBS cases [3] and approximately 1-2 individuals per million annually [4], and classically presents with the triad of ophthalmoplegia, areflexia, and ataxia. In rare cases, symptoms may progress and overlap with GBS. Beyond these classic signs, additional features can be associated, including ptosis in 60%, facial nerve palsy in 30%-50%, sensory deficits in 30%-50%, and hyposthenia in 20%-25% of cases [2].

MFS does not always present with the full triad. It can manifest as partial syndromes such as acute ophthalmoparesis, acute ataxic neuropathy, acute ptosis, acute mydriasis, or acute ataxic hypersomnolence [4].

Bickerstaff brainstem encephalitis is considered by some to be a variant of MFS, sharing features of ataxia and ophthalmoparesis but distinguished by decreased consciousness and hyperreflexia. Some authors classify it as a distinct GBS variant that frequently overlaps with MFS [2].

MFS is known to overlap with classic GBS in approximately 15% of MFS cases, which is itself a rare variant of GBS. MFS can also overlap with Bickerstaff encephalitis and pharyngeal-cervical-brachial GBS in approximately 35% of cases [5,6].

In MFS-GBS overlap syndrome, patients typically present with acute ophthalmoplegia, areflexia, and ataxia, followed by descending symmetrical limb weakness. In our patient, the initial asymmetry and proximal weakness obscured diagnostic certainty; however, absent reflexes and rapid progression ultimately revealed the diagnosis [4].

In our index case, flaccid quadriparesis preceded by ophthalmoparesis, which occurred in the absence of encephalopathy, making Bickerstaff brainstem encephalitis less likely. Myasthenia gravis was effectively excluded by the absence of fatigable weakness, electrophysiological findings inconsistent with a neuromuscular junction disorder, and the lack of clinical improvement following treatment with corticosteroids and pyridostigmine. Furthermore, the temporal evolution of symptoms, with ophthalmoparesis followed by progressive limb weakness culminating in flaccid quadriparesis, was more consistent with a Miller Fisher syndrome-Guillain-Barré syndrome (MFS-GBS) overlap variant.

Approximately two-thirds of all patients with GBS report some form of infection, either respiratory or gastrointestinal, in the weeks preceding the onset of weakness. Antecedent *Campylobacter jejuni* infection occurs in only approximately 20% of Miller Fisher cases, compared with over 75% in classic GBS-AIDP or AMAN, which may explain the absence of a preceding diarrhoeal illness in our patient. Approximately 8% of cases follow *Haemophilus influenzae* infection, and various viral associations have also been reported [7], including respiratory tract infections such as COVID-19. Our patient had experienced a viral respiratory tract infection several weeks before presentation. The average interval from infection to onset of neurological symptoms is approximately seven days. CSF analysis in GBS typically demonstrates albuminocytological dissociation. Anti-GQ1b antibodies are present in more than 85% of Miller Fisher cases, although they may be absent in overlap syndromes. Other antiganglioside antibodies, including anti-GT1, anti-GM1, and anti-GD1b, have also been identified in MFS. A limitation of this case report was that anti-GQ1b antibodies were not tested for, as this was not available within the institution.

Electrophysiological findings in MFS are variable, often showing sensory axonal involvement with relatively preserved motor conduction, whereas overlap syndromes more commonly demonstrate mixed motor abnormalities. The most consistent electrodiagnostic abnormality in MFS is the absence of the H-reflex, reflecting impairment of neuromuscular spindle fibres that express the GQ1b epitope. This also accounts for the areflexia and ataxia characteristic of MFS.

The ganglioside GQ1b is highly expressed on oculomotor nerves and muscle spindles, which explains the ocular involvement, absent reflexes, and sensory ataxia characteristic of Miller Fisher syndrome. Although the ataxia in GBS has been attributed to cerebellar dysfunction in some cases, this is not well substantiated. GQ1b is also thought to play a role in stabilising paranodal formation [8].

Immunopathogenesis of Guillain-Barré syndrome

There are several interrelated steps underlying the immunopathology of GBS and its variants. These include molecular mimicry, breakdown of the blood-nerve barrier, activation and proliferation of autoreactive T and B lymphocytes, cytokine release, and production of antiganglioside antibodies. These antibodies mediate target recognition and complement activation with membrane attack complex (MAC) formation, ultimately resulting in demyelination, axonal injury, and macrophage-mediated clearance of neural debris [9,10].

Trigger and molecular mimicry

GBS is typically initiated by an immune trigger, most commonly an antecedent infection, particularly *Campylobacter jejuni* [9]. Certain strains of *Campylobacter jejuni* express lipo-oligosaccharides (LOS) that share structural homology with peripheral nerve gangliosides such as GM1, GD1a, and GQ1b. Through molecular mimicry, antigen-presenting cells process and present these ganglioside-like epitopes, leading to a breakdown of immune tolerance and activation of autoreactive T lymphocytes and B lymphocytes [10,11].

Immune amplification and disruption of the blood-nerve barrier

Following immune activation, autoreactive T cells release pro-inflammatory cytokines, including interferon-γ, tumour necrosis factor-α, and interleukins, which induce endothelial activation and the expression of adhesion molecules and matrix metalloproteinases. These processes compromise the integrity of the blood-nerve barrier, allowing circulating immune cells and antibodies access to the endoneurial compartment [12]. Concurrently, B cells differentiate into antibody-secreting plasma cells, amplifying the autoimmune cascade. Breakdown of the blood-nerve barrier represents a pivotal step enabling immune-mediated peripheral nerve injury [10].

Peripheral nerve immune response

Within the peripheral nervous system, autoreactive T cells undergo reactivation and clonal expansion following antigen recognition. These cells differentiate into T helper 1 (Th1), T helper 2 (Th2), and T helper 17 (Th17) subsets. Th1 cells promote macrophage activation through interferon-γ and tumour necrosis factor-α secretion, while Th2 cells support B-cell proliferation and sustained autoantibody production. A relative reduction in regulatory T-cell activity further perpetuates the inflammatory milieu [9].

Target recognition and complement-mediated tissue injury

Autoantibodies generated during the immune response bind selectively to neural targets based on the distribution of gangliosides within peripheral nerves. Antibodies directed against GM1 and GalNAc-GD1a preferentially bind to motor axolemmal membranes at the nodes of Ranvier, resulting in predominantly motor deficits, as seen in axonal variants of GBS [13].

Antibodies against GQ1b localise to structures enriched in ocular motor pathways, muscle spindles, and the brainstem reticular formation, accounting for ophthalmoplegia, ataxia, and altered consciousness. Anti-GT1a antibodies target gangliosides expressed in lower cranial nerves, leading to bulbar dysfunction [10]. Antibody binding activates the complement cascade, culminating in deposition of complement components and MAC formation, which disrupts axonal and Schwann cell membranes [11].

Resultant effects: Demyelination, axonal injury, and nodopathies

Complement activation and macrophage-derived inflammatory mediators, including reactive oxygen species and proteolytic enzymes, result in segmental demyelination and nodal disruption. In axonal variants, primary axonal degeneration predominates [13].

Additionally, autoantibodies targeting nodal and paranodal proteins such as neurofascin-155 and contactin-associated protein-1 define a subgroup of immune-mediated neuropathies termed nodopathies. These are associated with distinctive clinical features including distal weakness, tremor, severe neuropathic pain, and relative resistance to conventional therapies [14,15].

Macrophage-mediated clearance and repair

Activated macrophages infiltrate peripheral nerves and remove damaged myelin, axonal fragments, and cellular debris through complement-dependent and Fc receptor-mediated mechanisms. Although excessive macrophage activation may exacerbate nerve injury, this process is essential for debris clearance and creates a permissive environment for remyelination and functional recovery [9].

Pathogenesis unique to Miller Fisher syndrome

The ganglioside GQ1b is highly expressed on oculomotor nerves and muscle spindles, explaining the classical triad of ophthalmoplegia, areflexia, and sensory ataxia seen in MFS. Although cerebellar dysfunction has been proposed as a mechanism for ataxia in GBS, this remains unsubstantiated. GQ1b plays a stabilising role at paranodal regions of peripheral nerves [8].

Molecular mimicry underlies the immune-mediated attack on nerve roots and heavily myelinated fibres, leading to albuminocytologic dissociation in cerebrospinal fluid and, in some cases, positive antiganglioside antibodies. Nerve conduction studies performed after the first week typically demonstrate conduction block and loss of H-reflexes [10].

Management

Prompt recognition of GBS and its variants is critical, as early initiation of immunomodulatory therapy significantly improves outcomes. Disease-modifying treatment should be offered to patients with non-ambulatory disease, rapidly progressive weakness, bulbar involvement, or respiratory compromise [9].

Immunomodulatory therapy

First-line treatment consists of either intravenous immunoglobulin (IVIG) at a dose of 0.4 g/kg/day for five consecutive days or plasma exchange (PLEX), typically 4-6 exchanges over 1-2 weeks. Both therapies are equally effective when initiated within the first two weeks of symptom onset, although IVIG is often preferred due to ease of administration and lower complication rates [10]. Combining IVIG and PLEX offers no additional benefit and is not recommended.

A large prospective study involving patients with MFS demonstrated a 100% survival rate, with comparable outcomes between IVIG-treated patients (0.4 g/kg/day for five days) and those receiving plasma exchange (average of four cycles). In terms of speed of neurological improvement, IVIG appeared to confer a modest advantage [10].

Supportive and critical care

Supportive management is equally crucial and includes close monitoring of respiratory function with serial forced vital capacity measurements, early admission to a high-dependency or intensive care setting when indicated, and prevention of complications such as aspiration pneumonia, autonomic instability, venous thromboembolism, pressure ulcers, and neuropathic pain. Early multidisciplinary rehabilitation, including physiotherapy, occupational therapy, and speech and language therapy, is also vital [1].

Prognosis

Overall prognosis in GBS and particularly MFS is favourable, with most patients achieving substantial recovery. However, patients with MFS-GBS overlap syndromes may experience a disease course more akin to classic GBS, with slower recovery and increased morbidity [9].

## Conclusions

This case highlights the dangers of anchoring bias in neurological diagnosis and underscores the importance of repeated clinical reassessment. While history remains central to neurological diagnosis, patient experience can colour symptom interpretation. Objective neurological signs, particularly areflexia in the context of acute quadriparesis, should prompt consideration of peripheral neuropathy or polyradiculopathy, including Guillain-Barré syndrome.
